# Transforming growth factor-β promotes aggressiveness and invasion of clear cell renal cell carcinoma

**DOI:** 10.18632/oncotarget.9177

**Published:** 2016-05-04

**Authors:** Raviprakash T. Sitaram, Pramod Mallikarjuna, Maréne Landström, Börje Ljungberg

**Affiliations:** ^1^ Department of Surgical and Perioperative Sciences, Urology and Andrology, Umeå University, Umeå, Sweden; ^2^ Department of Medical Biosciences, Pathology, Umeå University, Umeå, Sweden

**Keywords:** ccRCC, ALK5, pSmad2/3, PAI-1, TGF-β signaling pathway

## Abstract

The molecular mechanisms whereby transforming growth factor-β (TGF-β) promotes clear cell renal cell carcinoma (ccRCC) progression is elusive. The cell membrane bound TGF-β type I receptor (ALK5), was recently found to undergo proteolytic cleavage in aggressive prostate cancer cells, resulting in liberation and subsequent nuclear translocation of its intracellular domain (ICD), suggesting that ALK5-ICD might be a useful cancer biomarker. Herein, the possible correlation between ALK5 full length (ALK5-FL) and ALK5-ICD protein, phosphorylated Smad2/3 (pSmad2/3), and expression of TGF-β target gene PAI-1, was investigated in a clinical ccRCC material, in relation to tumor grade, stage, size and cancer specific survival. Expression of ALK5-FL, ALK5-ICD, pSmad2/3 and PAI-1 protein levels were significantly higher in higher stage and associated with adverse survival. ALK5-ICD, pSmad2/3 and PAI-1 correlated with higher grade, and ALK5-FL, pSmad2/3 and PAI-1 protein levels were significantly correlated with larger tumor size. Moreover, the functional role of the TGF-β - ALK5-ICD pathway were investigated in two ccRCC cell lines by treatment with ADAM/MMP2 inhibitor TAPI-2, which prevented TGF-β-induced ALK5-ICD generation, nuclear translocation, as well as cell invasion. The present study demonstrated that canonical TGF-β Smad2/3 pathway and generation of ALK5-ICD correlates with poor survival and invasion of ccRCC *in vitro*.

## INTRODUCTION

Renal cell carcinoma (RCC) accounts for about 90% of all tumors of the kidney [1] and includes different tumor types, classified according to their histology and specific gene alterations [2, 3]. The most common RCC type, clear cell RCC (ccRCC) is characterized by aberrations in chromosome 3p, usually including the loss of the VHL gene; while, other gene aberrations have been associated with tumor progression and metastatic behavior [4]. The TGF-β signaling pathway is involved in various cellular functions such as differentiation, proliferation, apoptosis, migration, and epithelial-mesenchymal transition (EMT) [5, 6]. The TGF-β exerts its functions through canonical (Smad-dependent) or non-canonical (non-Smad) TGF-β signaling pathway by regulating the TRAF6/TAK1, m-TOR, RhoA, Ras, MAPK, PI3K/AKT, PP2A/p70s6K, and JNK pathways [7–9]. In the canonical TGF-β pathway, TGF-β ligand binds to a constitutively active TGF-β type II receptor (TβRII); then, TβRII forms a heterotetrameric complex with a TGF-β type I receptor (TβRI, ALK5, ALK5-Full Length or ALK5-FL), and activates it by phosphorylation. Active ALK5-FL phosphorylates the receptor-associated Smad proteins (R-Smad), an assembly that forms a complex with co-Smad (Smad4) and translocates to the nucleus to induce target genes [10–12]. The Smad3/Smad4 complex target genes carry the optimal binding sequence, GTCTAGAC, for the Smad Binding Element (SBE) [13]. The TGF-β-induced Smad3/Smad4 complex also binds to a CAGA box, which is half of the typical SBE. The CAGA box is localized in the promoters of PAI-1 [14], JunB [15], and Smad7 [16]. In the non-canonical TGF-β pathway, TGF-β activates the ubiquitin ligase tumor necrosis factor receptor (TNFR)-associated factor 6 (TRAF6) that ubiquitinates TβRI in a TRAF6 mediated Lys63-linked polyubiquitination manner. The polyubiquitination of TβRI leads to its selective proteolytic cleavage by TACE and Presenilin (PS1), which liberates the TβRI-intracellular domain (TβRI-ICD or ALK5-ICD) [17–19]. Then, ALK5-ICD translocates to the nucleus and associates with transcriptional co-activator p300, promoting tumor invasion by induction of target genes (SNAIL1, ZEB, and MMP2) [18, 20]. Nuclear localization of ALK5-ICD also promotes its own expression by binding to the TβRI promoter [18] and regulates cell cycle, differentiation, and invasiveness in prostate cancer through cyclin D1 (CCND1) [19]. The exact mechanism of tumor aggressiveness by this pathway is still unclear, and the role of TβRI-ICD has not yet been studied clinically in RCCs.

Plasminogen activator inhibitor type-1 (PAI-1) is a target of TGF-β signaling [21], and PAI-1, a serine protease, converts plasminogen to plasmin. Expression of PAI-1 in tumor cells is higher than in normal cells and is associated with tumor growth, invasion, and metastasis [22, 23]. The PAI-1 concentration in cells also increases angiogenesis [24]. In breast cancer, it has a pivotal role in tumor vascular remodeling and neovascularization [25]. Expression of PAI-1 promotes cancer progression and high levels of PAI-1 proteins are associated with poor survival in some malignancies [26–29]. In ccRCC, high PAI-1 expression is correlated with a high nuclear grade, an advanced stage, distant metastasis, and shortened disease-free survival [30, 31].

In this study, we investigated the role of TGF-β signaling in the progression and aggressiveness of ccRCC with emphasis on ALK5-FL, ALK5-ICD, pSmad2/3, and PAI-1. We determined the correlations of these proteins with each other and their association with clinicopathological parameters such as tumor stage, nuclear grade, and tumor size, as well as survival of the patients. To further evaluate our clinical findings that expression of ALK5-ICD correlated with poor survival, cell transfection experiments were performed in two different ccRCC cell lines to evaluate if TGF-β -induced generation of ALK5-ICD is linked to invasion of ccRCC *in vitro.*

## RESULTS

### Expression patterns of ALK5-FL, ALK5-ICD, pSmad2/3, and PAI-1 proteins and PAI-1 mRNA in ccRCC and kidney cortex tissues

**Figure 1:**
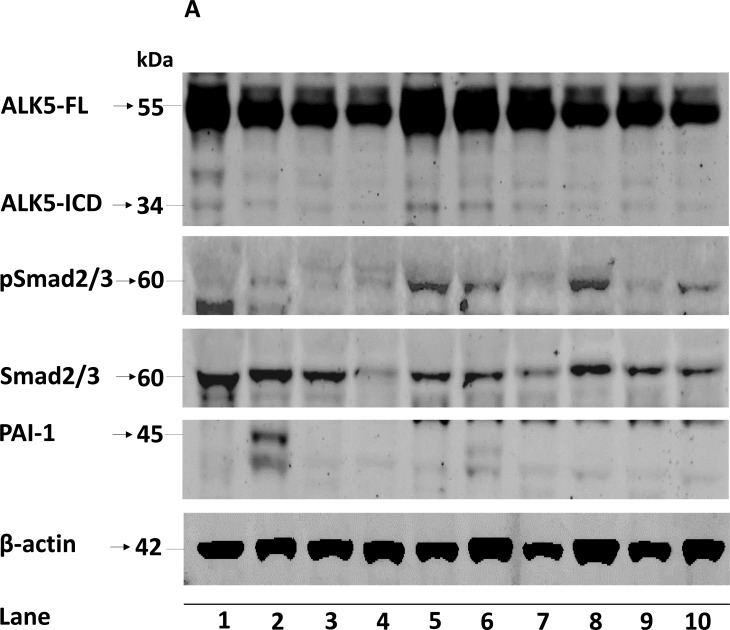

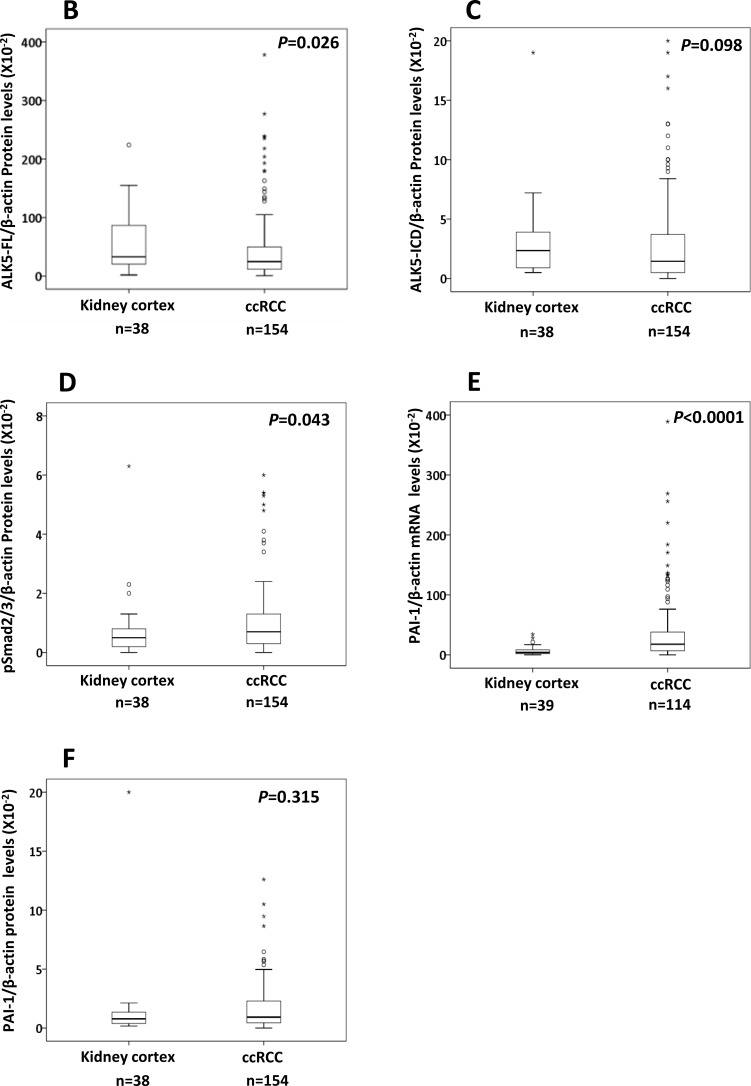
**A.** Representative immunoblots (10 out of 154 ccRCC tumor samples loaded in lane 1-10) showing expression of ALK5-FL, ALK5-ICD, pSmad2/3, total Smad2/3 and PAI-1 in ccRCC tissues. β-actin served as internal loading control; Box plot representation of expression of **B.** ALK5-FL, **C.** ALK5-ICD, **D.** pSmad2/3, **E.** PAI-1 mRNA, and **F.** PAI-1 protein in the kidney cortex compared with ccRCC tumors (Significant at *P* < 0.05, Mann-Whitney U-test).

The ALK5-FL protein was significantly lower (*P* = 0.026) in ccRCC (*n* = 154) compared with kidney cortex tissues (*n* = 38); while, ALK5-ICD was not different (*P* = 0.098). Expression of the pSmad2/3 protein was significantly higher (*P* = 0.043) in tumors (*n* = 154) than the kidney cortex tissues (*n* = 38). Expression of PAI-1 mRNA was also significantly higher (*P* < 0.0001) in ccRCC (*n* = 114) than the kidney cortex tissues (*n* = 39). Expression of PAI-1 protein was not significantly different (*P* = 0.315) (Figure 1). When considering only tumor samples that also had corresponding kidney cortex tissues (*n* = 36), ALK5-ICD was significantly lower in ccRCC (*P* = 0.005), and PAI-1 mRNA was significantly higher in ccRCC (*P* < 0.0001, *n* = 39). There was no difference for ALK5, pSmad2/3, and PAI-1 proteins (Supplementary Figure S1).

### Expression of ALK5-FL, ALK5-ICD, pSmad2/3, and PAI-1 proteins and PAI-1 mRNA and their relation with clinicopathological parameters

Protein levels of ALK5-FL, ALK5-ICD, pSmad2/3, and PAI-1 did not differ according to age or sex (data not shown). Expression of ALK5-FL was significantly associated with tumor stage and size; while, there was no difference in nuclear grade (Table 1). Expression of ALK5-ICD was significantly higher in advanced tumor stage and in higher nuclear grade but not in tumor size (Table 1). Protein levels of pSmad2/3 and PAI-1 were higher in advanced tumor stage, higher nuclear grade, and larger tumors (Table 1), The levels of PAI-1 mRNA did not correlate with tumor stage, tumor grade and size (Table 2). Furthermore, both PAI-1 mRNA (Table 2) and PAI-1 protein levels (Table1) were significantly higher in samples from patients with metastatic ccRCC compared with non-metastatic ccRCC.

**Table 1:** Relation between protein levels of ALK5-FL, ALK5-ICD, pSmad2/3, and PAI-1 and clinicopathological parameters

	ALK5-FL			ALK5-ICD			pSmad2/3			PAI-1		
Parameter	*n*^a^	Mean Rank	*P*^b^	*n*^a^	Mean Rank	*P*^b^	*n*^a^	Mean Rank	*P*^b^	*n*^a^	Mean Rank	*P*^b^
**Grade**												
I	76	74.1		76	70.34		76	69.9		76	64.41	
			0.35			0.049			0.036			<0.0001
II	78	80.81		78	84.48		78	84.9		78	90.26	
**TNM Stage**												
Early Stage												
(I+II)	84	69.4		84	66.34		84	68.17		84	63.17	
			0.014			0.001			0.004			<0.0001
Advanced Stage												
(III+IV)	70	87.22		70	90.89		70	88.7		70	94.7	
**Metastasis**												
M0	114	74		114	71.25		114	70.1		114	70.1	
			0.1			0.003			<0.0001			0.001
M1	40	87.49		40	95.31		40	98.59		40	98.59	
**Tumor Size (mm)**												
<70	77	69.42		77	71.22		77	68.92		77	67.73	
			0.024			0.08			0.017			0.007
>70	77	85.58		77	83.78		77	86.08		77	87.27	
												

a Number of patients

b Groups were compared using Mann-Whitney U-test (Significant at *P* < 0.05)

**Table 2:** Relation of PAI-1 mRNA levels with clinicopathological parameters

	PAI-1 mRNA		
Parameter	*n*^a^	Mean Rank	*P*^b^
**Grade**			
I	57	57.32	
			0.955
II	57	57.68	
**TNM Stage**			
Early Stage			
(I+II)	63	52.78	
			0.090
Advanced Stage			
(III+IV)	51	63.33	
**Metastasis**			
M0	83	53.65	
			0.042
M1	31	67.81	
**Tumor Size (mm)**			
<70	60	54.33	
			0.280
>70	54	61.03	

a Number of patients

b Groups were compared using Mann-Whitney U-test (Significant at *P*<0.05)

### Correlations between ALK5-FL, ALK5-ICD, pSmad2/3, and PAI-1 proteins and PAI-1 mRNA levels

Protein levels of ALK5-FL, ALK5-ICD, pSmad2/3, and PAI-1 significantly correlated with each other (Table 3). In addition, PAI-1 mRNA levels positively correlated with pSmad2/3 (Pearson correlation = 0.450, *P* < 0.0001, *n* = 101) and PAI-1 protein levels (Pearson correlation = 0.218, *P* = 0.028, *n* = 101), but not ALK5-FL and ALK5-ICD.

**Table 3:** Association between ALK5-FL, ALK5-ICD, pSmad2/3 and PAI-1 protein levels in ccRC samples

Variables	ALK5-ICD	pSmad2/3	PAI-1
**ALK5-FL**	*P* < 0.000^‡^, *r* = 0.637 *n* = 154	*P* < 0.000^‡^, *r* = 0.332 *n* = 154	*P* < 0.000^‡^, *r* = 0.453 *n* = 154
**ALK5-ICD**		*P* < 0.000^‡^, *r* = 0.354 *n* = 154	*P* < 0.000^‡^, *r* = 0.511 *n* = 154
**pSmad2/3**			*P* < 0.000^‡^, *r* = 0.488 *n* = 154

‡ Determined using Spearman correlation (Significant at *P* < 0.05)

### Protein levels of ALK5-FL, ALK5-ICD, pSmad2/3, and PAI-1 and PAI-1 mRNA levels and their relation with cancer specific survival

**Figure 2:**
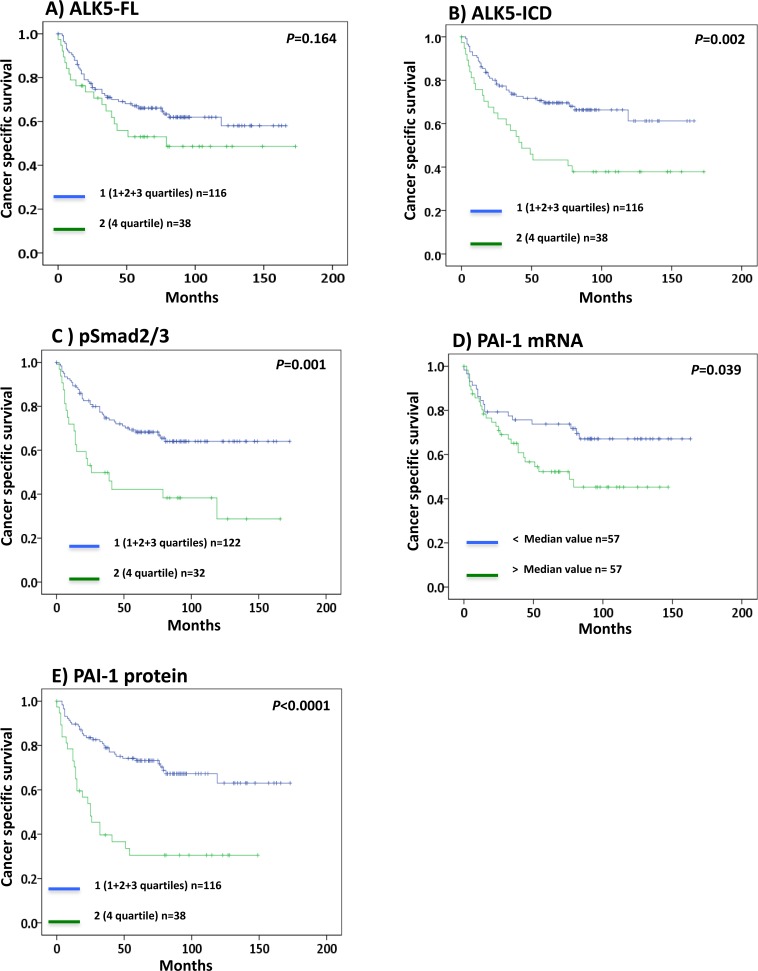
Kaplan-Meier plots showing cancer specific survival curves of **A.** ALK5-FL, **B.** ALK5-ICD, **C.** pSmad2/3, **D.** PAI-1 mRNA, and **E.** PAI-1 protein levels in ccRCC (Significant at *P* < 0.05).

In survival analysis, patients with ALK5-ICD (*P* = 0.002), pSmad2/3 (*P* = 0.001), and PAI-1 (*P* < 0.0001) protein levels in the upper 4^th^ quartile had significantly shorter survival compared with the lower levels (1^st^+2^nd^+3^rd^ quartiles) as shown in Figure 2. In contrast, ALK5-FL protein levels did not show any significance with survival (*P* = 0.164, Figure 2A). When dividing the patient according to the median value of these variables, no significant survival association remained, but PAI-1 mRNA levels showed significant survival association (*P* = 0.039, Figure 2D).

### *In vitro* ectopic overexpression of ALK5-HA in the ccRCC cell lines 786-0 and A498

**Figure 3:**
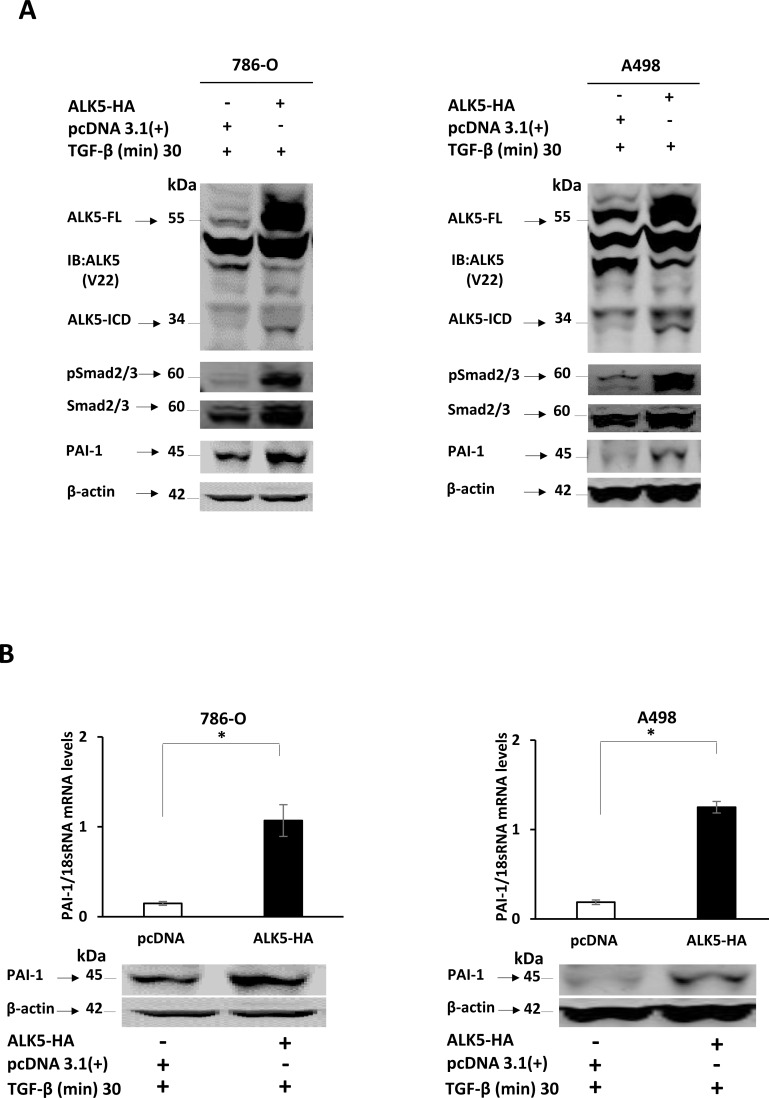

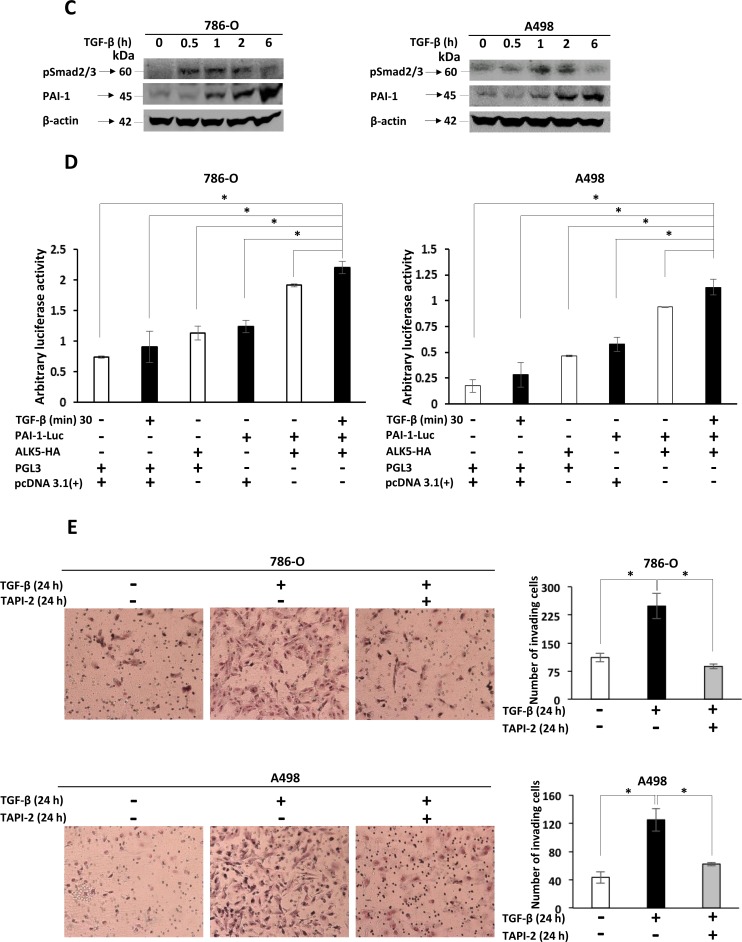
**A.** Immunoblots showing protein expression of ALK5-FL, ALK5-ICD, pSmad2/3, total Smad2/3 and PAI-1, in 786-O and A498 cells at 24 h after ectopic overexpression of the ALK5-HA vector or pcDNA3.1(+1) control, followed by treatment with TGF-β. β-actin served as internal loading control (n = 3 independent experiments). **B.** Levels of PAI-1 mRNA and protein after ectopic overexpression of ALK5-HA in ccRCC cell lines. β-actin served as internal loading control (mean ± s.d., *P < 0.05, Student's t-test, *n* = 3 independent experiments). **C.** Immunoblots showing protein expression of pSmad2/3, PAI-1 in 786-O and A498 cells at different time points after stimulation with TGF-β. β-actin served as internal loading control **D.** Luciferase assay showing luciferase activity in 786-0 and A498 cells co-transfected with PAI-1-Luc promoter and ALK5-HA, and stimulated with TGF-β for 30 min, compared with the cells co-transfected with empty PGL3 Vector and pcDNA 3.1(+), or empty PGL3 Vector and pcDNA 3.1(+), and stimulated with TGF-β for 30 min, or empty PGL3 vector and ALK5-HA, or PAI-1-Luc promoter and pcDNA 3.1(+) or PAI-1-Luc promoter and ALK5-HA (mean ± s.d., *P < 0.05, Student's t-test, *n* = 2 independent experiments). **E.** Invasion assay showing the invasiveness of 786-0 and A498 cells treated with TGF-β compared with untreated cells and with cells treated with TAPI-2 (TACE-inhibitor) along with TGF-β (quantification is represented graphically after counting invading cells, mean ± s.d., *P < 0.05, Student's t-test, *n* = 3 independent experiments).

To confirm that PAI-1 expression was associated with the TGF-β pathway *via* pSmad2/3, two different ccRCC cell lines, 786-0 and A498, were used for *in vitro* transfection with the ALK5-HA expression vector. Transient overexpression of ALK5-HA and treatment with TGF-β resulted in an increase in the protein expression of ALK5-FL and ALK5-ICD at 24 h compared with pcDNA (empty vector control). The ALK5-ICD protein was also increased in treated cells containing the ALK5-HA vector, and this was accompanied by increased phosphorylation of pSmad2/3, and PAI-1 mRNA and protein. These results indicated that ALK5-HA overexpression activated Smad2/3 by phosphorylation, which leads to increase in the expression of PAI-1 mRNA and protein levels (Figure 3A and 3B). The Immunoblot filters were probed with HA antibody to detect the HA-tagged proteins. The results demonstrated that the ALK5-ICD was derived from the transfected source ALK5-HA vector (Supplementary Figure S2).

### *In vitro* TGF-β1 stimulation of ccRCC cell lines

When the 786-0 and A498 cells were stimulated with TGF-β1 ligand, a time dependent and a significant increase in phosphorylation of Smad2/3 and PAI-1 protein expression was observed (Figure 3C). To investigate the invasive capacity induced by the TGF-β ligand, an invasion assay was performed using the 786-0 and A498 cell lines. A higher proportion of cells (2.5-fold for 786-0 cells and 3-fold for A498) crossed the matrigel in the presence of TGF-β when compared with untreated cells (Figure 3E). This result clarifies the invasive capacity imparted to cells by TGF-β. To further investigate the role of ALK5-ICD on the invasiveness of the cells, TAPI-2 (TACE-inhibitor) was added to the cell media along with TGF-β. The invasive capacity of both 786-O and A498 cell lines were relatively decreased (2.5-fold for 786-0 and 2-fold for A498), compared with TGF-β treated cells (Figure 3E). Thus, blocking the formation of ALK5-ICD by ALK5-FL cleavage leads to a decrease in cell invasion.

Immunoblot and Immunofluorescence experiments validated the invasion assay. The nuclear entrance of cleaved intracellular domain of endogenous ALK5-FL receptor driven by TGF-β in 786-O and A498 cells was reduced by TAPI-2 as shown in the supplementary Figure S3A, B.

### PAI-1 promoter activity analysis

To evaluate the ability of TGF-β-Smad pathway in regulating PAI-1 promoter activity, a luciferase assay was performed in 786-0 and A498 cell lines. Highest promoter activity (arbitrary luciferase activity) was seen in cells co-transfected with PAI-1-Luc promoter construct and ALK5-HA stimulated with TGF- β for 30 min when compared with the cells co-transfected with empty PGL3 Vector and pcDNA 3.1(+), or the cells co-transfected with empty PGL3 Vector and pcDNA 3.1(+), and stimulated with TGF-β for 30 min, or the cells co-transfected with empty PGL3 vector and ALK5-HA, or the cells co-transfected with PAI-1-Luc promoter and pcDNA 3.1(+), or the cells co-transfected with PAI-1-Luc promoter and ALK5-HA. This result indicated that PAI-1 promoter activity was transcriptionally triggered by the TGF-β-Smad pathway (Figure 3D).

## DISCUSSION

In this clinical study on patients with ccRCC, we present novel findings on the associations of canonical TGF-β-Smad pathway and generation of ALK5-ICD correlates with tumor progression, invasion, and poor prognosis.

The TGF-β is involved in various cellular processes such as homeostasis, fibrosis, angiogenesis, wound healing, and carcinogenesis [5, 6]. It also acts as a tumor suppressor in early stages of the tumor, and then switches to a tumor promoter in later stages [32–34]. Mutation or decreased expression of TβRII, and decreased expression of TβRI (ALK5-FL) have been reported in different types of cancers [8]. Our data in the present study are consistent with these results, and showed lower ALK5-FL protein levels in tumors compared with the kidney cortex, and simultaneously higher ALK5-FL protein levels in more advanced tumor stages and larger tumors. Our results confirm reports from earlier studies that the TGF-β signaling pathway has a role in tumor aggression in ccRCC, but the exact role was not clear [35, 36]. Earlier reports found there was altered VHL gene expression in ccRCC that associated with high expression of TGF-β and a bad prognosis [37, 38], but in this study ALK5-FL protein levels did not show any significant association with cancer-specific survival.

The canonical TGF-β pathway signals through the Smad complex [36]; therefore, we analyzed the expression of phosphorylated (activated) Smad2/3 (pSmad2/3) in ccRCC. Expression of the pSmad2/3 protein was significantly higher in ccRCC than in the kidney cortex, and was significantly associated with nuclear grade, tumor stage, and size. Thus, our results corroborate earlier studies, showing that high pSmad2 expression in a subset of tumors correlated with a poor prognosis [39–41]. In contrast, another ccRCC study showed a correlation between low pSmad2 expression and a bad prognosis [42]. The discrepancy is unclear, but might be due to differences in techniques and sampling. Our study also showed a significant correlation between pSmad2/3 and the other TGF-β pathway proteins analyzed. These findings were expected since pSmad2/3 is a component of the TGF-β signaling pathway and a known substrate of ALK5-FL [8].

We also analyzed PAI-1 mRNA and protein expression in ccRCC since it was previously shown that TGF-β induces expression of the PAI-1 gene *via* Smad [14, 19]. We found that PAI-1 mRNA levels were significantly higher in ccRCC than in the kidney cortex, in contrast to the PAI-1 protein levels. Previous studies have shown that PAI-1 expression was higher in various carcinomas than in normal cells [22, 23]. One probable reason for this difference in mRNA and protein data could be that PAI-1 mRNA (only at the transcriptional level) is synergistically regulated by TGF-β and epidermal growth factor (EGF) or other growth factors. The synergistic effect stabilizes PAI-1 mRNA levels and increases mRNA production, which occurs independently from *de novo* protein production. We showed that PAI-1 mRNA is highly expressed in advanced ccRCC, and PAI-1 protein is associated with advanced stage, larger tumors, and poor patient survival, confirming results from previous studies [43–45]. One earlier study on ccRCC using immunohistochemistry also reported that PAI-1 was associated with tumor stage and disease progression [31], which is in line with our findings. Furthermore, we found that high PAI-1 mRNA as well as PAI-1 protein levels were associated with poor survival of the patients, confirming the independent survival information previously reported [29, 30]. The association between PAI-1 and tumor progression might be due to the regulation of angiogenesis by PAI-1 [30]. Knockdown of PAI-1 substantially reduced angiogenesis in mice [30].

Previously our lab showed that a parallel pathway to the canonical signaling pathway is activated and TGF-β, *via* TRAF6, causes polyubiquitination of ALK5-FL and promotes its cleavage to ALK5-ICD by TNF-alpha converting enzyme (TACE), in a PKCζ-dependent manner [17]. The liberated intracellular domain (ICD) of ALK5-FL has a specific role in tumor invasion through regulation of the transcription of genes (SNAIL1, ZEB, and MMP2) involved and in association with the transcriptional regulator p300 to activate genes [17, 20]. Although, ALK5-ICD was localized in the nucleus of malignant prostate cells, bladder cancer cells, and RCC tissue sections, the exact mechanism of ALK5-ICD nuclear translocation is not well understood. It was also observed that the nuclear ALK5-ICD promoted its expression and cell invasion by altering cell cycle proteins like CCD1 [19]. Further, *in vivo* mice experiments showed that inhibition of ALK5-ICD cleavage prevented mouse prostate tumor growth [18]. Our study showed for the first time that ALK5-ICD has a role in tumor progression and metastasis in ccRCC. Since our study is the first to investigate ALK5-ICD protein expression in clinical tumor material, there are no ALK5-ICD protein levels to compare our results with. In addition, we showed that patients with high ALK5-ICD protein levels had significantly shorter cancer-specific survival times than those with lower levels. These novel results revealed the importance of ALK-ICD protein in the progression of ccRCC. These results are in line with previous studies of cancer cells showing nuclear localization of ALK5-ICD in clinical RCCs [17], and its role in invasiveness and cell proliferation [19].

Gene profiling analysis and experimental data indicated a poor prognosis of ccRCC, activation of TGF-β signaling, and evidence of Smad2 activation in clinical samples [46]. In the present clinical study, all the canonical TGF-β-signaling pathway proteins (ALK5, pSmad2/3, and PAI-1) studied significantly correlated with each other. These TGF-β pathway proteins all associated with tumor progression, metastasis, and survival showing that the active canonical TGF-β pathway is important in the progression of ccRCC. Although, the formation, translocation to nucleus, and functions of ALK5-ICD occur independently from the canonical TGF-β pathway, ALK5-ICD was associated with proteins involved in the pathway. A probable explanation for this finding might be auto-regulation of ALK5-ICD. Binding to its own promoter (ALK5) in the nucleus [18, 19] might form a positive feedback loop to activate the canonical TGF-β pathway. Integration of both canonical and non-canonical pathways at several levels [18] may be the underlying mechanism for the positive correlation observed [19].

ALK5-HA was overexpressed in the ccRCC cell lines, 786-0 and A498, and we observed increased expression of pSmad2/3 protein, PAI-1 mRNA, and PAI-1 protein. On the endogenous level, stimulation of the 786-O and A498 cell lines with the TGF-β ligand activated ALK5 and further promoted the TGF-β/Smad/PAI-1 pathway. A luciferase assay showed that the PAI-1 promoter activity was increased after overexpression of ALK5 followed by stimulation of TGF-β. Invasion assay demonstrated that the invasive capacity of ccRCC cells increased after TGF-β stimulation. The invasive capacity after TGF-β stimulation was decreased when ALK5-ICD formation was blocked by a TACE inhibitor (TAPI-2). These results are consistent with a previous study [18]. These *in vitro* data from the present study are in agreement with previous reports that the TGF-β/Smad complex transcriptionally activates PAI-1 [19] and promotes cancer progression and invasiveness.

In conclusion, our study demonstrated that the TGF-β-Smad signaling pathway significantly promotes tumor progression, metastasis, and survival in ccRCC. We also report the novel finding that ALK5-ICD is positively associated with TGF-β-Smad signaling and has a key role in tumor progression, metastasis, and survival. The canonical pathway might increase the aggressiveness and angiogenesis of ccRCC through its transcriptional target PAI-1. The exact molecular mechanism involved in the translocation of ALK5-ICD to the nucleus, the association of ALK5-ICD with the canonical Smad pathway, and the role of PAI-1 in tumor progression and metastasis should be investigated further.

## MATERIALS AND METHODS

### Patient samples

The study included 154 patients (90 men and 64 women) diagnosed with ccRCC and surgically treated between 2000 and 2009. The mean age was 65.9 years (range 32-87 years), and the median tumor size was 70 mm (range 12-190 mm). For mRNA studies, the number of samples used depended upon availability and samples from 114 patients (47 men and 67 women) with a mean age of 64.32 years (range 32-85 years) and mean tumor size of 70 mm (range 10-190) were analyzed. Samples from the tumor and kidney cortex tissue from the tumor bearing kidney were immediately obtained at the time of nephrectomy using a viable area for sampling, as described previously [47]. Each sample was divided, and one part was snap frozen immediately and stored at −80°C until analysis. The other part was formalin fixed for histological confirmation of the tumor as well as non-malignant kidney cortex. For protein levels, kidney cortex tissues were analysed for 38 out of 154 patients, and 39 out of the 114 patients for mRNA. Kidney cortex tissues are corresponding non-malignant kidney cortex obtained from the same kidney from which tumor was obtained. These kidney cortex tissues were pathologically determined to be non-cancerous. The samples were collected after obtaining signed consent from the patients. The study was approved by the institutional review board and the ethical committee of Northern Sweden. Tumor stage was determined according to the TNM classification system 2002 [48], and the clinical stage was evaluated by physical examination and computerized tomography of the chest and abdomen. There were 59 patients in TNM stage I (38.3%), 25 patients in stage II (16.2%), 30 patients in stage III (19.5%), and 40 patients in stage IV (26.0%). For mRNA there were 47 patients in TNM stage I (41.2%), 16 patients in stage II (14.0%), 20 patients in stage III (17.5%), and 31 patients in stage IV (26.0%). Nuclear grade was determined according to Fuhrman et al [49]. For statistical use, stage I and stage II were merged in one group, and stage III and stage IV in another group. Similarly, grade I and II were merged in one group, and grade III and grade IV in another group. Eighteen tumors were classified as grade 1 (11.7%), 58 tumors as grade 2 (37.7%), 51 tumors as grade 3 (33.1%), and 27 tumors as grade 4 (17.5%). The RCC type was classified according to the Heidelberg consensus conference [2]. For mRNA, 13 tumors were classified as grade 1 (11.4%), 44 tumors as grade 2 (38.6%), 36 tumors as grade 3 (31.6%), and 21 tumors as grade 4 (18.4%). Vein invasion was verified by microscopic examination of renal hilus tissue slides and was defined as the presence of tumor-derived thrombi in the major renal veins. Capsule invasion was defined as invasion of tumor cells through the perinephric or renal sinus fat. Tumor size was defined as the maximum diameter determined by CT. Follow-up medical reports of the patients were used for survival analysis. Patients were followed in accordance with a scheduled program. At the last follow-up, 61 (39.6%) patients were alive without any indications of disease, 6 (3.9%) were alive with disease, 60 (39.0%) died due to the disease, and 27 (17.5%) died due to other reasons. For mRNA, 43 (37.7%) patients were alive without any indications of disease, 5 (4.4%) were alive with disease, 45 (39.5%) died due to the disease, and 21 (18.4%) died due to other reasons. The survival status was last updated on 26-05-2015.

### Protein extraction and analysis

Protein was extracted from the clinical samples by slightly mincing the tissue using a surgical knife and then adding TRAPeze^®^ 1X CHAPS Lysis Buffer (EMD Millipore, Billerica, MA, USA). The samples were incubated on ice and placed on a shaker for 30 min. For the cells collected from *in vitro* studies, TRAPeze^®^ 1X CHAPS Lysis Buffer was added, and samples were incubated on ice for 30 min with frequent vortexing. The samples were then centrifuged at 14000 rpm for 30 min. The supernatant, which consisted of proteins, was carefully extracted. Nuclear and cytoplasmic proteins were separated, and extracted using abcam nuclear extraction kit (ab113474, abcam, Cambridge, MA, USA) following the manufacturer's instructions. Protein estimation was done using the Thermo Scientific™ Pierce™ BCA™ Protein Assay Kit (Thermo Fisher Scientific, Waltham, MA, USA) following the manufacturer's protocol.

### Immunoblot (IB)

Proteins were separated on NuPAGE^®^ Novex^®^ 7% Tris-Acetate Protein Gels (Life Technologies, Carlsbad, CA, USA) or NuPAGE^®^ Novex^®^ 10% Bis-Tris Protein Gels using the XCell SureLock™ Mini-Cell (Life Technologies). All wells were loaded with 30 μg of proteins. Separated proteins were then transferred onto Trans-Blot^®^ Turbo™ Midi Nitrocellulose membranes (Bio-Rad Laboratories, Hercules, CA, USA) using the Trans-Blot^®^ Turbo™ Transfer Starter System (Bio-Rad Laboratories) at 2.5 V for 15 min. Membranes were blocked in Odyssey^®^ Blocking Buffer (Licor Biosciences, Lincoln, NE, USA), diluted in an equal volume of 1x Tris-Buffered Saline for 60 min at room temperature. The membrane was then probed with the following antibodies: TGF-β RI (V-22) or ALK5 diluted 1:250 (sc-398, Santa Cruz Biotechnology, Santa Cruz, CA, USA), a specific antibody that identifies ALK5-FL and ALK5-ICD [17, 18], HA diluted 1:1000 (CST #2367, Cell signaling technology, Danvers, MA, USA), phospho-Smad2 diluted 1:500 (CST #3108), Smad2 diluted 1:1000 (CST #3122), PAI-1/Serpine1 diluted 1:1000 (NBP1-19773, Novus Biologicals, Littleton, CO, USA), Lamin A/C diluted 1:1000 (CST #2032), β-tubulin diluted 1:1000 (CST #2128) and β-actin diluted 1:10000 (A5316, Sigma-Aldrich St. Louis, MO, USA). The primary antibodies were detected using either IRDye^®^ 800CW Goat anti-Rabbit (Licor #926-32211, Licor Biosciences) or IRDye^®^ 680RD Goat anti-Mouse (Licor #925-68070, Licor Biosciences). The membranes were visualized at 84 μm in an Odyssey^®^ CLx Infrared Imaging System (Licor Biosciences) and analyzed using Image Studio™ Software version 3.1 (Licor Biosciences). The relative levels (numerical density value) for all proteins were calculated by dividing their numerical density value by that of the housekeeping protein, β-actin.

### RNA and cDNA extraction

Total RNA was isolated from the snap frozen tissue samples using TRIzol^®^ (Life Technologies). The RNA concentrations were measured spectrophotometrically at 260/280 nm (NanoDrop 1000 Spectrophotometer, Thermo Fisher Scientific).

cDNA was made using the High-Capacity cDNA Reverse Transcription Kit (Life Technologies), which contains 10X RT Buffer, 10X RT Random Primers, 25X dNTP Mix (100mM) MultiScribe^®^ Reverse Transcriptase (50U/μL). RNAase inhibitor (life technologies) was used. The protocol used according to the manufacturer's manual.

### Real-time PCR

Quantitative real-time polymerase chain reaction (qRT-PCR), using Applied Biosystems 7900HT Fast Real-Time PCR System (Applied Biosystems, Foster City, CA, USA), was performed for PAI-1 mRNA expression. The Primer/Probe Set Hs01126606_m1 (Applied Biosystems) for PAI-1, Hs99999901_s1 (Applied Biosystems) for 18sRNA, and for β-actin sequence of primer/probe as previously described [50] was used. The relative mRNA levels of PAI-1 were normalized to housekeeping gene β-actin (clinical study) and 18sRNA (*in vitro* study), and given in relation to expression of PAI-1 in calibrators A498 (*in vitro* study) and 786-O (clinical study) using the delta-delta method [51].

### Cell culture and *in vitro* studies

The 786-O and A498 RCC cell lines were bought from ATCC in 2012. Prior to experiments, both cell lines were authenticated by STR profiling (Identicell, Denmark) on 12-06-2014, and were cultured in RPMI media supplemented with 10% FBS.

One day prior to transfection, 1 × 10^6^ cells (786-O and A498) were seeded in a 10 cm plate. To synchronize the cell cycle, cells were starved by adding Opti-MEM and incubating at 37°C for 15 min. Transient transfection of the cell lines with 20 μg C-terminally hemagglutinin (HA)-tagged ALK5 (ALK5-HA) [17] or 20 μg pcDNA 3.1(+) (control) was done using Lipofectamine 3000 reagent (Life Technologies) according to manufacturer's protocol. Cells were collected the next day after stimulation with 10 ng/ml TGF-β1 (R&D Systems, Minneapolis, MN, USA) for 30 minutes. For the kinetic experiment, cells (786-O and A498) stimulated with 10 ng/ml TGF-β1 were collected at 0 min, 30 min, 1 h, 2 h, and 6 h. For immunoblot experiments, the cells were treated with 10 ng/ml TGF-β or 10 ng/ml TGF-β and 20 μM TAPI-2 (Enzo life sciences, Farmingdale, NY, USA) or untreated cells were collected after 6 h.

### Luciferase assay

Reporter vectors containing the PAI-1-Luc promoter with the CAGA binding site were generated using BGLII and HindIII (Fermentas, Life Technologies) sites. The primers used for PCR amplification were 5′-TCG AGA GCC AGA CAA GGA GCC AGA CAA GGA GCC AGA CAC- ′3 and 3 ′-AGC TCT CGG TCT GTT CCT CGG TCT GTT CCT CGG TCT GTG-5′. Sequencing was carried out to verify all the constructs. One day prior to transfection, 1 × 10^6^ cells (786-O and A498) were seeded in a 96-well plate. The cells were then transiently co-transfected with 0.2 μg of internal control Renilla and 0.2 μg each of empty PGL3 Vector (control for PGL3-PAI-1-Luc) and pcDNA 3.1(+) (Control for ALK5-HA), or empty PGL3 Vector and pcDNA 3.1(+), and stimulated with TGF-β for 30 min or empty PGL3 vector and ALK5-HA, or PGL3 vector containing the PAI-1-Luc promoter and pcDNA 3.1(+), or PGL3 vector containing the PAI-1-Luc promoter and ALK5-HA, or PGL3 vector containing the PAI-1-Luc promoter and ALK5-HA, and stimulated with TGF-β for 30 min; using Lipofectamine 3000 (Life Technologies). Cells were collected after 24 h of transfection and analyzed by luciferase assay using the Dual-Luciferase^®^ Reporter Assay System (Promega, Fitchburg, WI, United States). A representative transfection experiment was performed in duplicate and luciferase values shown in the figures are from at least two independent experiments.

### Invasion assay

The invasion assay and analysis was performed for 786-O and A498 cell lines using a Corning^®^ BioCoat™ Matrigel^®^ invasion chamber (catalog #354480, Corning Incorporated, Corning, NY, USA) following the manufacturer's instructions. The starved 786-0 and A498 cells were stimulated with 10 ng/ml TGF-β1 or 10 ng/ml TGF-β1 and 20 μM TAPI-2 for 24 h, untreated cells served as controls. Invasiveness was assessed after fixing the cells in 3.7% formaldehyde for 2 min, followed by incubation in 100% methanol for 20 min for cell permeabilization. Cells were stained using Giemsa stain and visualized under a Leitz light microscope (Germany) at magnification 10X. Images were captured using DpxViewPro software (Denmark).

### Immunofluorescence and confocal image

Immunofluorescence assay was performed as described from the earlier study [19]. In brief, two-well chamber slides were (Sigma-Aldrich) seeded with 786-O and A498 cells (5*10^5^ cells/ well). Next day cells were stimulated with 10 ng/ml TGF-β1 or 10 ng/ml TGF-β1 and 20 μM TAPI-2 for 6 h. Then the cells were washed with 1x PBS, fixed with 3.7% formaldehyde (10 min), and permeabilized with 2% triton-X (10 min). Cells were blocked using 5% Bovine Serum Albumin. After blocking, cells were incubated with the primary antibody ALK5 (V-22) antibody (diluted 1:50) for 1 h followed by secondary antibody Alexa Fluor^®^ 555 anti-rabbit (Catalog #A-31572, Life Technologies). The slides were mounted using VECTASHIELD^®^ HardFSet™ Mounting Medium with DAPI (Catalog# H-1500, Vector Laboratories, Burlingame, CA, USA) according to manufacturer's instructions. Confocal Images were taken by Zeiss 710 meta (Carl Zeiss MicroImaging, Inc.) digital camera (RET-EXi-F-M-12-C) by using 63X 1.4/ NA magnification lens. Images were captured using ZEN 2010 software.

### Statistical analysis

All statistical analysis was performed using IBM SPSS Statistics 22.0. Bivariate correlations between two proteins were analyzed using Spearman and Pearson correlations. The Mann-Whitney U-test was used to analyze the difference in expression of two independent variables. Kaplan-Meier curves were used to express survival times, and the log-rank test was used to compare the survival times. The Student's t-test was used to compare the mRNA expression in cell culture, invasion of cells, arbitrary luciferase activity, and Immunofluorescence. For all the tests, a *P* value less than 0.05 was considered significant.

## SUPPLEMENTARY MATERIAL FIGURES


