# Supplementary material for: Transforming growth factor-β promotes aggressiveness and invasion of clear cell renal cell carcinoma
Source: Oncotarget. 2016 May 4;7(24):35917–31. doi: 10.18632/oncotarget.9177 (PMC5094972; doi:10.18632/oncotarget.9177)
Supplement: Supplementary file 1 [file oncotarget-07-35917-s001.pdf]

Transforming growth factor-β promotes aggressiveness and invasion of clear cell renal cell carcinoma

Supplementary Material

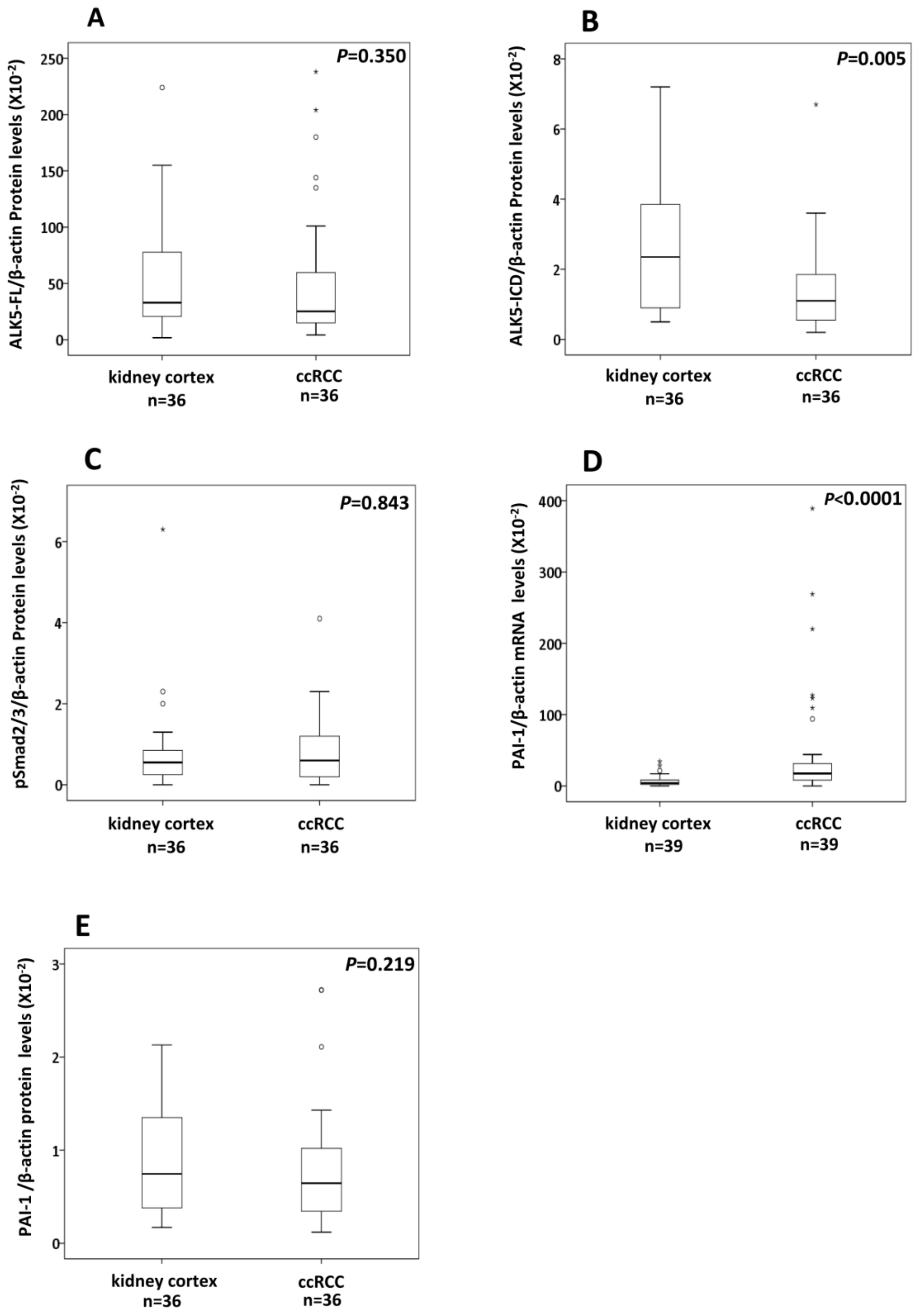

Supplementary Figure S1: Box plot representation of expression of (A) ALK5-FL, (B) ALK5-ICD, (C) pSmad2/3, (D) PAI-1 mRNA, and (E) PAI-1 protein in ccRCC tumors compared with corresponding non-malignant tissue from the same kidney (Significant at $P<0.05$, Mann-Whitney U-test).

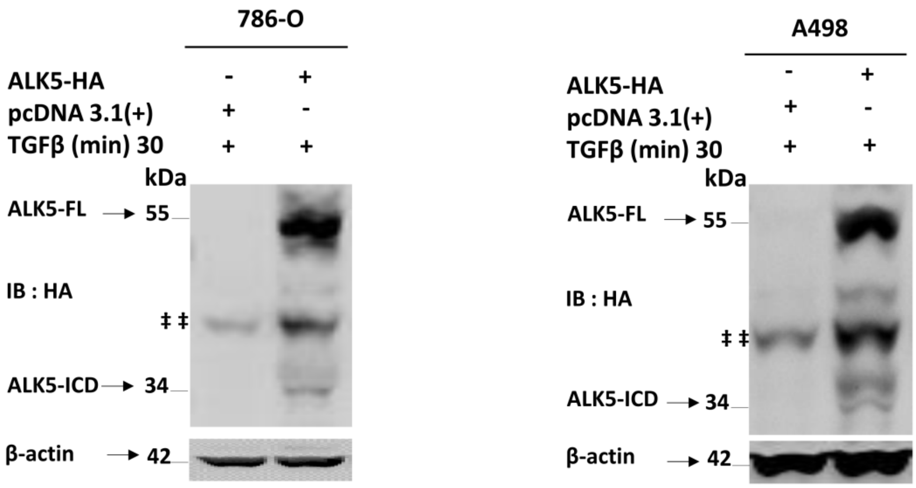

Supplementary Figure S2: Immunoblot showing protein expression of HA-tagged ALK5-FL (55 kDa) and ALK5-ICD (34 kDa) in 786-O and A498 cells at 24 h after ectopic overexpression of the ALK5-HA vector or pcDNA3.1(+1) control, followed by treatment with TGF-β. β-actin served as internal loading control (‡‡ represents a background band).

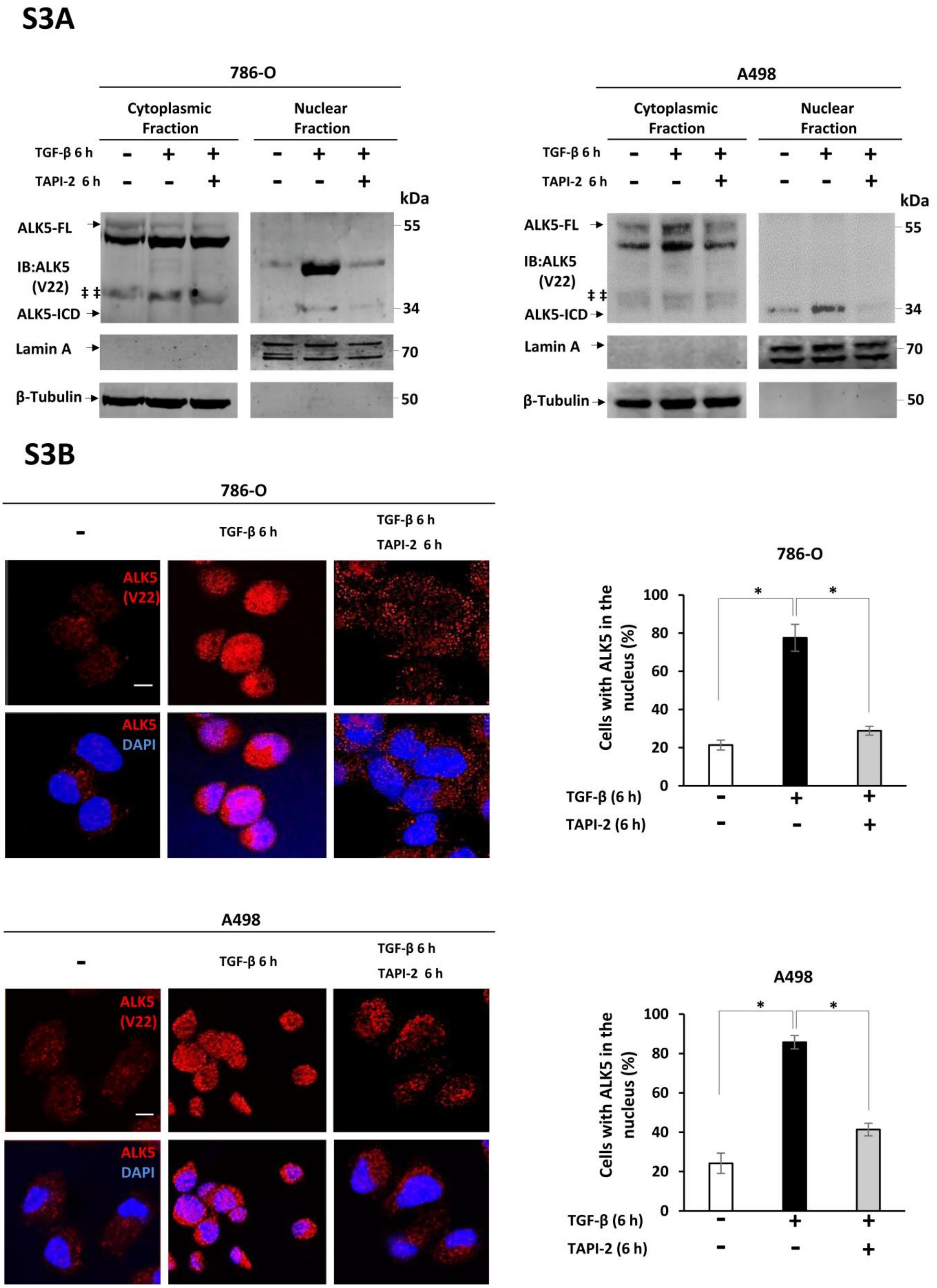

Supplementary Figure S3: (A) Immunoblots showing cytoplasmic and nuclear protein expression of ALK5-FL, ALK5-ICD, in 786-0 and A498 cells treated with TGF-β for 6 h compared with untreated cells and with cells treated with TAPI-2 along with TGF-β for 6 h (‡‡ represents a background band). Lamin A and β-tubulin served as internal loading controls for nuclear extract and cytoplasmic extract respectively. (B) Immunofluorescence of endogenous ALK5 visualized using ALK5 (V22) antibody in 786-0 and A498 cells after

cells treated with TGF-β for 6 h compared with untreated cells and with cells treated with TAPI-2 along with TGF-β for 6 h. Cell nuclei were stained with DAPI. Scale bar 20 μm (quantification is represented graphically after measuring 200-250 cells, mean ± s.d., *P<0.05, Student's t-test, n=3 independent experiments).
